# Meta-analysis of selective laser trabeculoplasty versus topical medication in the treatment of open-angle glaucoma

**DOI:** 10.1186/s12886-015-0091-2

**Published:** 2015-08-19

**Authors:** Xingyi Li, Wei Wang, Xiulan Zhang

**Affiliations:** Zhongshan Ophthalmic Center, State Key Laboratory of Ophthalmology, Sun Yat-Sen University, 54S.Xianlie Road, Guangzhou, 510060 China

## Abstract

**Background:**

The aim of this study was to examine possible differences in clinical outcomes between selective laser trabeculoplasty (SLT) and topical medication in the treatment of open-angle glaucoma.

**Methods:**

Pertinent prospective comparative controlled trials comparing SLT with medication were selected through extensive searches of the PubMed, Embase, Web of Science, Chinese Biomedicine Database, and the Cochrane Controlled Trials Register database from their inception up to March 2014. Efficacy estimates were measured by their weighted mean difference (WMD) to calculate the intraocular pressure reduction (IOPR) from baseline to endpoint and by the odds ratio (OR) to determine success rates.

**Results:**

Five prospective studies, which met the predefined criteria, were included in the meta-analysis. Four studies were randomized clinical trials and one study was a prospective non-randomized clinical trial. There were a total of 492 eyes of 366 patients with open-angle glaucoma. Four studies involving 325 eyes compared SLT with medication in terms of the IOPR. The WMD of the IOPR from the baseline was 0.6 (95 % confidence intervals: −0.24,1.43) when comparing SLT with medication. No statistical heterogeneity was observed between studies (χ2 = 1.30, P = 0.75, I^2^ = 0.0 %). All five studies reported success rates, with a pooled OR of 0.84 (95 % confidence intervals: 0.42, 1.68), which was not statistically significant. No statistical heterogeneity was observed between studies (χ2 = 5.98, P = 0.200, I^2^ = 33.1 %). Subgroup and sensitivity analysis confirmed the high stability of the meta-analysis results.

**Conclusions:**

Both SLT and topical medication demonstrate similar success rates and effectiveness in lowering intraocular pressure in patients with open-angle glaucoma.

**Electronic supplementary material:**

The online version of this article (doi:10.1186/s12886-015-0091-2) contains supplementary material, which is available to authorized users.

## Backgrond

Glaucoma is a leading cause of blindness worldwide [1]. It is estimated that more than 60 million people had glaucoma in 2010, 8.4 million of whom are bilaterally blind as a result of the disease.There are 79.6 million people around the world who will have glaucoma by 2020, and 74 % of them will have open-angle glaucoma (OAG) [2]. Lowering the intraocular pressure (IOP) is still the goal of OAG treatment and remains the most effective way to prevent the development and progression of glaucoma. Currently, there are three methods available to achieve this goal: medication, laser treatments, and surgery [3].

Medication therapy is typically the first approach for reducing IOP. Currently, many kinds of potently hypotensive topical medicines are available for controlling IOP [4]. However, medications have potential disadvantages. Patients must tolerate drug side effects, repeated application of drugs and ongoing medical costs [5]. The introduction of selective laser trabeculoplasty (SLT) provided a new non-invasive choice for the reduction of IOP in eyes with OAG [6]. This treatment consists of the application of laser spots in the trabecular meshwork which leads to an increase in the outflow facility and, consequently, decreases IOP.

In a previous Cochrane systematic review on laser trabeculoplasty, only one study comparing SLT with medication was included, which prevented the meta-analysis at that time [3]. In a recent meta-analysis comparing SLT with argon laser trabeculoplasty (ALT) in the treatment of OAG, the role of medication also was not evaluated [7]. Thus,the effectiveness of SLT compared to contemporary medication requires further investigation. Recently, McAlinden et al. [8] reviewed the IOP-lowering effect when comparing SLT to other glaucoma treatment options. In that article, the author only described the effects of SLT and did not perform a meta-analysis.

Currently, several published clinical trials have compared the efficacy and safety of SLT with medical therapy [9–13]. For example, Lai et al. [13] reported that the IOP-lowering effect of SLT in Chinese patients is comparable with topical medications. However, these studies had modest sample sizes and conveyed inconclusive results. To date, no consensus has been reached on this topic. Previously, we conducted a meta-analysis comparing SLT with ALT; the results reveal that SLT has similar efficacy to ALT with a similar constellation of side effects [14]. To our knowledge, the data comparing SLT with topical medication in the treatment of OAG has not been systematically evaluated and reported. Therefore, we conducted a systematic review and meta-analysis of all published prospective clinical trials to assess the outcomes of SLT versus medication in the treatment of OAG.

## Methods

The Preferred Reporting Items for Systematic Reviews and Meta-Analyses (PRISMA) statement was used as a guide to conduct the study, including the strategies for searching, analysis, the presentation of results, potential bias, interpretation, and writing (Additional file 1: Table S1) [15].

### Literature search and trial selection

Studies were identified and retrieved through a systematic search of PubMed, Embase, the Web of Science, the Chinese Biomedicine Database, and the Cochrane Controlled Trials Register database from their inception up to March 2014. Periodical literature update was performed and the most recent literature retrieving was at June 30, 2015. However, no new original articles comparing SLT to medication was identified. Keywords used in identifying relevant researches included ‘selective laser trabeculoplasty’, ‘Yag laser trabeculoplasty’, ‘selective laser trabeculectomy’, ‘Nd:YAG’, ‘SLT’, ‘glaucoma’, and ‘ocular hypertension’. Searches were performed by combining the keywords or their relevant abbreviations and truncations. No restriction was applied for language or year of publication. The websites of professional associations and Google Scholar were also searched for additional information. Moreover, a manual search was performed by checking the reference lists of all retrieved trials to identify studies not yet included in the computerized databases. Eligible studies were prospective randomized or non-randomized comparative controlled trials, which compared the use of SLT and topical anti-glaucoma medications in adult patients with any form of naïve OAG or ocular hypertension (OHT).

### Data extraction and outcome measures

 The information on author, age, sex, country, duration, sample size, type of glaucoma, study design, and other outcome data were extracted.  The incidence of transient post-laser IOP spikes, sustained IOP elevation, anterior chamber reaction, discomfort, redness, and pain were recorded. For studies with overlaps of population, the latest one were included in the final analysis. Given that the effectiveness of OAG treatment is often assessed by the intraocular pressure reduction (IOPR) and percent of intraocular pressure reduction (IOPR %), the primary efficacy outcomes were the IOPR and IOPR % from the baseline to the end of follow up. The secondary efficacy outcome was the success rate. Success was defined as IOPR ≥ 3 mm Hg and/or IOPR % ≥20 % [7]. The adverse events in each intervention were also reviewed.

### Assessment of methodology quality

The Downs and Blacks scale, which could evaluate both randomized and non-randomized studies, were used for assessment of the clinical trials [16]. The system comprises 27 items distributed between five subscales regarding reporting (10 items), external validity (three items), bias (seven items), confounding (six items), and power (one item). Any discrepancy in the qualitative assessment between the two observers was discussed and a consensus was reached. The total score of each trial was expressed as a percentage of the maximum achievable score. Studies' methodological quality was assessed as excellent, good, fair, or poor when the total score was ≥20, from 15 to 19, from 11 to 14, and ≤10, respectively [17].

### Statistical analysis

The intent-to-treat analyses were used in each outcome. Additionally, data was combined using an inverse variance random-effects model regardless of heterogeneity [18].The weighted mean difference (WMD) was calculated for continuous outcomes while the odds ratio (OR) was estimated for dichotomous outcomes. All results were given with 95 % confidence intervals (CIs). Heterogeneity was checked using Cochran's Q statistic and the P-value. I^2^ metrics, which quantify heterogeneity irrespective of the number of studies, were also reported [19]. Studies with an I^2^ value of greater than 50 % were considered significant heterogeneity. A P*-*value of less than 0.05 was considered statistically significant. The statistical analysis was performed using Stata SE 12.0 (Stata Corporation LP, College Station, TX, USA).

### Sensitivity analysis and publication bias

We also performed subgroup analysis in terms of the medications used within the medication group. In addition, we investigated the influence of a single study on the overall pooled estimate by omitting one study in each turn. To detect publication biases, we visually examined asymmetry in funnel plots. Furthermore, the Begg’s and Egger’s measures were calculated.

## Results

### Literature search

**Fig. 1:**
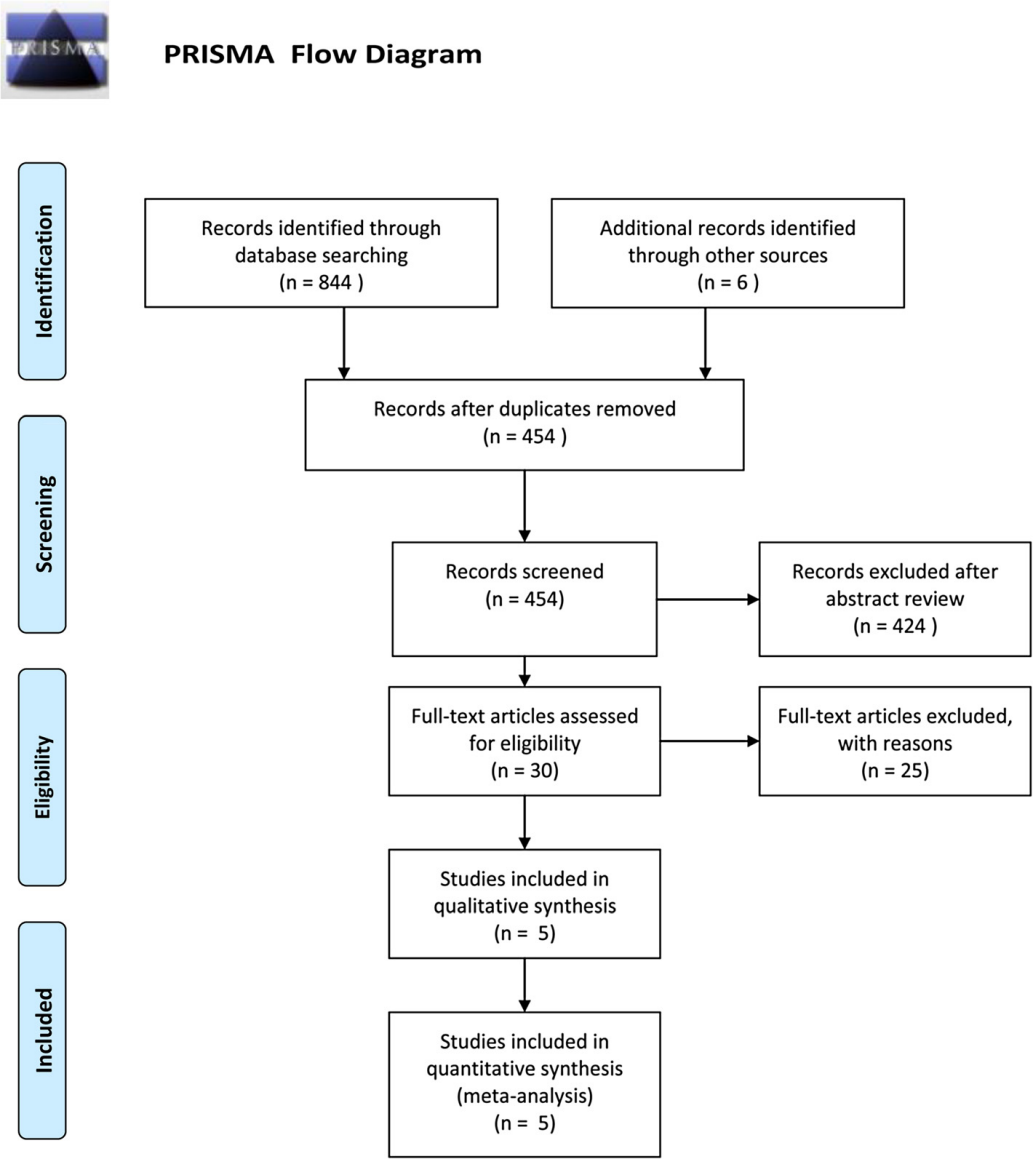
The selection flowchart of clinical trials included in this meta-analysis

The initial search yielded 850 relevant publications, of which 814 were excluded for being duplicates from multiple databases or for various other reasons (unrelated topic,reviews, case reports, or case series) on the basis of the titles and abstracts. The remaining 30 were retrieved for full-text review, and 25 of them were excluded because they were studies comparing SLT with other laser therapies rather than drugs (Additional file 2: Table S2). Hence, a final total of five studies published from 2004–2012 were included in this meta-analysis [9–13]. Fig. 1 provides a flow diagram of the search results.

### Characteristics and quality assessment of the included studies

In total, there were 492 eyes from 366 patients included in this meta-analysis; 318 eyes were included in the SLT group and 174 eyes were included in the medication group. The characteristics of the included studies are summarized in Table 1. The age of patients ranged from 25–82 years. One study was a prospective non-randomized comparative trial [11] and four studies were randomized clinical trials (RCT) [9, 10, 12, 13]. The studies had the following geographic distribution: one in the USA, one in China, one in Canada, and two in the UK. The mean duration of follow-up ranged from 4–60 months. In the studies included in the present meta-analysis, SLT was usually performed at 360° with an average power setting between 0.2 mJ and 1.7 mJ. The mean baseline IOP ranged from 25.0 mmHg to 29.3 mmHg in SLT groups, and from 22.8 mmHg to 29.0 mmHg in medication groups. Our prespecified definition of success was target IOP, but different and heterogeneous definitions were used in studies, such as IOP < 21 mmHg after intervention in one study [13], at least a 20 % IOP reduction from baseline measurement in three studies [10–12], and meet the target IOP in one study [9]. Table 2 shows the quality evaluation of each clinical trial. The Downs and Blacks score for each study exceed 16, which indicate adequate methodological quality.

**Table 1:** Characteristics of prospective comparative controlled trials comparing SLT with topical medication

Author(year)	Design, location	NO. of eyes (SLT/med)	Follow-up	Mean age	Baseline IOP	BCVA C/D	Diagnosis	Definition of success	Degrees of SLT/ Average energy	Medication
Lai(2004)	SC,RCT, China	29/29	60 m	51.9	26.8 ± 5.6 /26.2 ± 4.2	0.1-1.0/ 0.2-1.0	POAG, OHT	IOP ≤ 21 mmHg	360	Topical beta-blocker, pilocarpine, dorzolamide latanoprost as monotherapy or in combination
						0.4 ± 0.2/ 0.5 ± 0.2			1.0 ± 0.1 mJ	
Nagar(2005)	MC,RCT, UK	128/39	12 m	63	29.3/29	NA	POAG, OHT, PDS, PEX	IOPR% ≥ 20 %	360/180/90	Latanoprost 0.005 %
									0.2-1.7 mJ	
McIlraith(2006)	MC,Pro, Canada	74/26	12 m	62	26/24.6	NA	POAG, OHT, PDS, PEX	OP ≤ 22 mmHg	180	Latanoprost 0.005 %
						0.5 ± 0.2/ 0.6 ± 0.2			0.8 mJ	
Nagar(2009)	SC,RCT, UK	20/20	4-6 m	NR	26.1 ± 4.0 /22.8 ± 4.5	NA	POAG, OHT	IOPR% ≥ 20 %	360	Latanoprost 0.005 %
									0.2-1.4 mJ	
Katz(2012)	MC,RCT, USA	67/60	9-12 m	NR	25.0 ± 2.2 /24.5 ± 2.2	NA	POAG, PEX, OHT	Arrived target IOP	360 followed by 180 0.2-1.2 mJ	Topical prostaglandin analog, β-blocker, brimonidine, carbonic anhydrase inhibitor, in combination

Abbreviations: SLT = selective laser trabeculoplasty; M/F = male/female; m = month; NA = not available. SC = single center, MC = multi-center, RCT = randomized controlled trial; Pro = prospective non-RCT; intervention = SLT/medication; BCVA = best corrected visual acuity; C/D = cup to disc ratio; POAG = primary open angle glaucoma; OHT = ocular hypertension; PDS = pigment dispersion syndrome; PEX = pseudoexfoliation syndrome

**Table 2:** Quality scoring components for five clinical trials included in the meta-analysis

	Quality score component					Score	
First Author (year)	I	II	III	IV	V	Over all	Percentage (%)
Lai(2004)	11	2	5	3	3	24	75.00 %
Nagar(2005)	11	3	4	3	3	24	71.88 %
McIlraith(2006)	9	2	4	2	2	19	59.38 %
Nagar(2009)	11	3	4	3	3	24	71.88 %
Katz(2012)	11	3	4	3	3	24	71.88 %

I = reporting; II = external validity; III = bias (seven items); IV = confounding; V = power

### IOP reduction

**Fig. 2:**
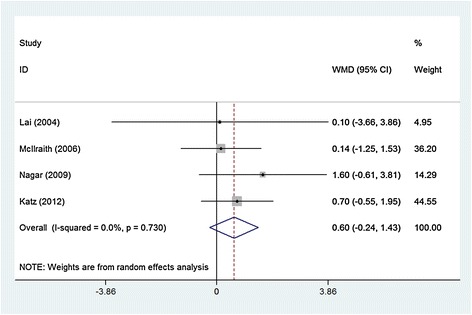
Comparison of intraocular pressure reduction from the baseline between SLT group and topical medication group. WMDs indicate weighted mean difference, which were computed by using a random-effects model

Four studies involving 325 eyes compared SLT with medication in terms of the IOPR (Fig. 2). No statistical heterogeneity was observed between studies (χ2 = 1.30, P = 0.75, I^2^ = 0.0 %). SLT was found to archive a numerically greater IOPR from baseline to end-point; however, the differences in the IOPR were not all statistically significant (WMD = 0.6, 95 % CI: −0.24, 1.43) (Table 3). We divided the studies into 3 subgroups according medication, and then performed a sensitivity analysis excluding the non-randomized study.

**Table 3:** Subgroup analysis of IOP reduction from the baseline and success rate comparing SLT with medication

Group	No. of studies	WMD/OR			Test for Heterogeneity			Test for Overall Effect	
		Estimate	Lower	Up	χ2	I^2^	P	Z	P
All	4	0.60	−0.24	1.43	1.30	0.00 %	0.730	1.40	0.163
Only RCT	3	0.86	−0.19	1.90	0.65	0.00 %	0.723	1.60	0.110
Combined medication	2	0.64	−0.55	1.83	0.09	0.00 %	0.767	1.05	0.292
Latanoprost-only	2	0.61	−0.73	1.94	1.20	16.4 %	0.274	0.89	0.374
**IOPR%**									
All	4	−1.90 %	−5.00 %	1.10 %	3.78	20.50 %	0.287	1.23	0.220
Only RCT	3	−3.20 %	−9.10 %	2.70 %	3.77	47.00 %	0.152	1.06	0.290
Combined medication	2	−0.80 %	−3.20 %	1.50 %	0.00	0.00 %	0.988	0.67	0.502
Latanoprost-only	2	−5.10 %	−13.50 %	3.20 %	2.45	59.2 %	0.117	1.20	0.230
All	5	0.84	0.42	1.68	5.98	33.1 %	0.200	0.50	0.621
Only RCT	4	0.85	0.34	2.11	5.98	49.8 %	0.113	0.35	0.726
Combined medication	2	1.46	0.61	3.49	0.23	0.00 %	0.629	0.85	0.396
Latanoprost-only	3	0.57	0.23	1.38	2.85	29.9 %	0.240	1.26	0.209

**Fig. 3:**
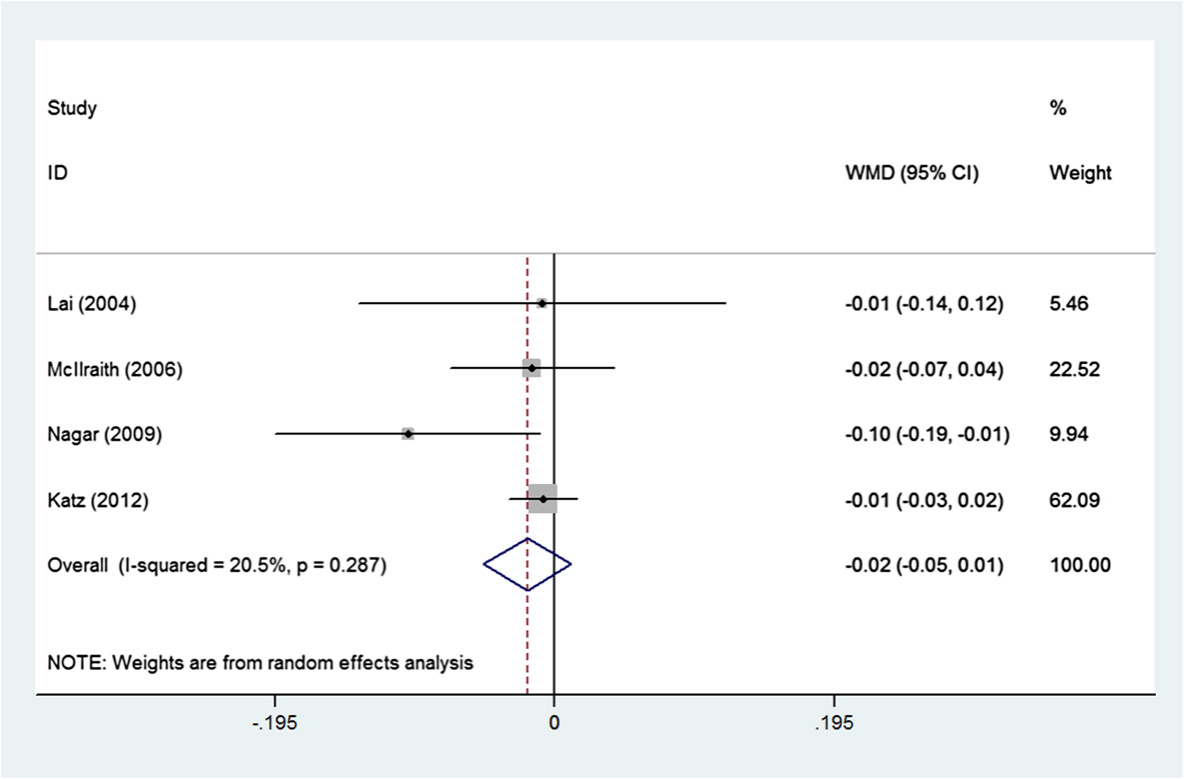
Comparison of percent intraocular pressure reduction from the baseline between SLT group and topical medication group. WMDs indicate weighted mean difference, which were computed by using a random-effects model

IOP = intraocular pressure; SLT = selective laser trabeculoplasty; IOPR = intraocular pressure reduction; IOPR %  = percent intraocular pressure reduction from baseline; Pro = prospective nonrandomized; RCT = prospective randomized controlled trial; OR = odds ratio;

All subgroups showed that SLT was similar to medication in IOPR (Table 3). Four studies reported the IOPR % at follow-up endpoint (Fig. 3). Pooling the results revealed no significant difference between the two groups, with a WMD for the IOPR % comparing SLT with medication of −1.9 % (95 % CI: −5.00 %, 1.10 %) (Table 3). When only including the RCTs, the differences in IOPR % were also statistically non-significant (Table 3). Additionally, there was no significant heterogeneity in these analyses. Among the biases in the studies, the unit of analysis issue should be mentioned because there were 492 eyes of 366 patients, which lead to overestimate precision of effect estimates since within-patient correlation is not accounted for.

### Success rate

**Fig. 4:**
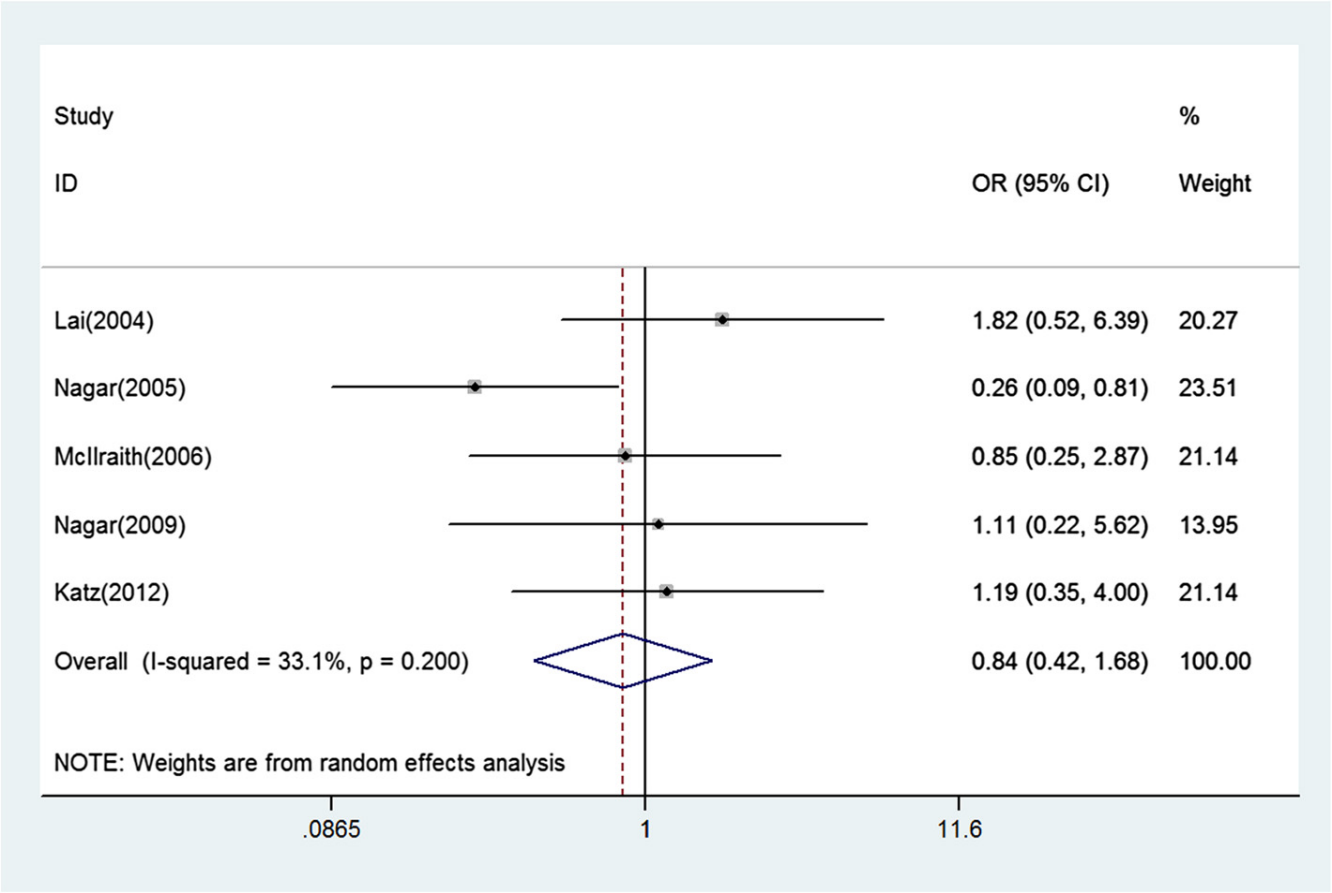
Comparison of success rates between SLT group and topical medication group. ORs indicate odds ratios, which were computed by using a random-effects model

Five studies reported the proportions of patients achieving target endpoint IOP at follow-up endpoint; the difference in success rate between the SLT group and the medication group was not statistically significant (pooled OR 0.84, 95 % CI: 0.42, 1.68) (Fig. 4). No statistical heterogeneity was observed between studies (χ2 = 5.98, P = 0.200, I^2^ = 33.1 %).For the subgroup, including RCTs, the difference in success rate between the SLT group and the medication group was not statistically significant either (OR 0.85, 95 % CI: 0.34, 2.11). For the subgroup, including studies using latanoprost, the difference in success rate was not statistically significant (OR 0.57, 95 % CI: 0.23,1.38) (Table 3).

### Adverse events

Concerning adverse events, three studies reported the types and incidences. However, we did not perform a meta-analysis because there were so few cases.In general, adverse events were transient and minor. Transient post-laser IOP spikein some patients was observed. There were no patients with sustained IOP elevation after SLT. Other common side effects, namely, anterior chamber reaction, discomfort, redness, and pain were also described as transient and without sequelae in all studies.

### Sensitivity analysis and publication bias

The sensitivity analyses suggested that  each study did not significantly influence the global estimation of  the IOPR, IOPR %, and success rate (Table 4). Due to the limited number (<10) of studies included in each analysis, publication bias was not assessed. Table 5 summarized the overall evidence of SLT versus medicine in the treatment of OAG.

**Table 4:** Results of leave-one-out sensitivity analyses

Study Excluded	Pooled WMD/OR			Test for Heterogeneity			Test for Overall Effect	
	Estimate	Lower	Up	χ2	I^2^	P	Z	P
**IOPR**								
Lai(2004)	0.62	−0.237	1.48	1.22	0.00 %	0.542	1.42	0.156
McIlraith(2006)	0.86	−0.193	1.90	0.65	0.00 %	0.723	1.60	0.110
Nagar(2009)	0.43	−0.48	1.33	0.37	0.00 %	0.829	0.93	0.353
Katz(2012)	0.51	−0.61	1.64	1.25	0.00 %	0.536	0.89	0.371
**IOPR%**								
Lai(2004)	−2.50 %	−6.60 %	1.60 %	3.77	46.90 %	0.152	1.18	0.237
McIlraith(2006)	−3.20 %	−9.10 %	2.70 %	3.77	47.00 %	0.152	1.06	0.290
Nagar(2009)	−0.90 %	−3.10 %	1.30 %	0.06	0.00 %	0.970	0.83	0.408
Katz(2012)	−3.90 %	−9.60 %	1.70 %	2.65	24.40 %	0.266	1.37	0.170
**Success rate**								
Lai(2004)	0.68	0.33	1.43	4.02	25.4 %	0.259	1.01	0.311
Nagar(2005)	1.20	0.63	2.29	0.74	0.00 %	0.864	0.54	0.588
McIlraith(2006)	0.85	0.34	2.11	5.98	49.8 %	0.113	0.35	0.726
Nagar(2009)	0.81	0.35	1.87	5.83	48.6 %	0.120	0.50	0.620
Katz(2012)	0.78	0.33	1.85	5.53	45.7 %	0.137	0.57	0.568

CI = confidence interval; IOPR = intraocular pressure reduction; IOPR% = percent intraocular pressure reduction from baseline

**Table 5:** Summary of finding table of the overall quality of evidence according to GRADE

Outcomes	Eye (Study)	SLT	Med	Absolute effect (95 % CI)	Relative effect (95 % CI)	Quality of evidence	Recommend
IOPR	325(4)	190	135	0.60 (−0.24, 1.43)	-	B	Low
IOPR%	325(4)	190	135	−1.90 % (−5.00 %,1.10 %)	-	B	Low
Success rate	298(4)	175	123	0.84 (0.42,1.68)	-	C	Low

## Discussion

Introduced in 1995 by Park and Latina [20], SLT provided a new choice for the reduction of IOP in eyes with OAG. SLT is easy to perform and well tolerated by patients.However, the efficacy and safety of SLT versus topical medications for OAG remain unclear. In the preliminary published studies, SLT was used as an adjunct therapy to medication [21]. Later, several studies suggested that SLT may serve as the primary therapy for OAG [22–26]. Comparisons of SLT and medication were done in the remaining trials, but there is still not enough evidence to determine which is the best choice [8].

In this meta-analysis, we reviewed five prospective comparative controlled trials. The results reveal that SLT is as effective as medication in regard to the control of IOP, which is consistent with a previous review [8]. Also, no heterogeneity was observed across the studies.In addition, SLT and medication are similar in their success rates. The results from our subgroup and sensitivity analyses were quite similar and robust. 

The exact mechanisms of SLT lowering IOP are not known. There are three prevailing theories, namely, the mechanical theory, the biologic theory, and the cell theory [27, 28] .According to the mechanical theory, contraction and shrinkage of trabecular beams caused by SLT exerts a pull on surrounding beams, which opens up the intertrabecular spaces.The biologic theory proposes that laser energy causes tissue injury with a resultant cascade of events. Macrophages are attracted and alter the secreted extracellular matrix, allowing an increased aqueous outflow.The cellular theory suggests that SLT applications stimulate cell division in the anterior trabecular meshwork, providing pluripotent cells for the repopulation of sites with laser therapy. These cells produce different extracellular matrices, enhancing the aqueous outflow. Additionally, there may be other mechanisms of action of SLT. It has been reported that exposure to factors secreted by lasered Schlemm canal cells and lasered trabecular meshwork cells and the application of prostaglandin analogs induced junction disassembly while increasing the permeability of Schlemm canal cells [29]. Recently, Chen et al. [30] determined the effect of travoprost (a drug similar to latanoprost) on Schlemm’s canal in healthy human eyes using fourier-domain optical coherence tomography. They observed that travoprost induced the expansion of Schlemm’s canal lumen. These findings suggest that SLT and latanoprost might share a common mechanism that likely mediated their similar IOP lowering effects.

The results of this meta-analysis must be interpreted cautiously in light of the strengths and limitations of the included trials. A key strength is the fact that all the studies included in this meta-analysis were published by established centers of excellence using a prospective comparative controlled design and all of them were well-performed and of high quality. Despite our rigorous methodology, some limitations of the current study should not be ignored. First, we cannot fully exclude publication bias. The number of included studies is insufficient to carry out statistical testing to detect publication bias. In addition, we did not attempt to gain access to unpublished results. Second, our analyses of IOPR, IOPR %, and success rate were based on data pooled from trials of different durations. This was due to the lack of data reported in all phases of follow-up and may have a potential impact on our results.Third, when discussing treatment result, it is important to realize that the criteria used to define success varies between studies. Differences between studies may also exist with respect to the diagnostic criteria for glaucoma and the severity of the disease. Fourth, none of the identified studies provided a cost-effectiveness analysis; thus, a prospective randomized clinical trial comparing the cost of SLT to medication is required. The effectsof SLT on other outcome measuressuch as delaying the need for surgery in people with early glaucoma were not assessed. This may be an interesting focus for future studies.

## Conclusions

In conclusion, the present meta-analysis suggests that SLT appears to be similar to medication in lowering intraocular pressures in patients with open angle glaucoma. Despite our rigorous methodology, the inherent limitations of the included studies should be considered, and conclusions drawn from our pooled results should be interpreted with caution. Future large-volume, well-designed RCTs with extensive follow-up are awaited to confirm and update the findings of this analysis.

## Additional files

Additional file 1: Table S1. PRISMA Checklist. (DOC 69 kb) Additional file 2: Table S2. The studies were excluded after full-text review. (DOCX 25 kb)
