# Supplementary material for: Meta-analysis of selective laser trabeculoplasty versus topical medication in the treatment of open-angle glaucoma
Source: BMC Ophthalmol. 2015 Aug 19;15:107. doi: 10.1186/s12886-015-0091-2 (PMC4544808; doi:10.1186/s12886-015-0091-2)
Supplement: Additional file 2: Table S2. — The studies were excluded after full-text review. (DOCX 25 kb) [file 12886_2015_91_MOESM2_ESM.docx]

**Table S2.** The studies were excluded after full-text review.

| **NO.** | **Authors** | **Title** | **Source** | **Reason** |
| --- | --- | --- | --- | --- |
| 1 | Kent SS, et al. | A Randomized Clinical Trial of Selective Laser Trabeculoplasty Versus Argon Laser Trabeculoplasty in Patients With Pseudoexfoliation. | J Glaucoma 2013. | SLT versus SLT |
| 2 | Rosenfeld E, et al. | The efficacy of selective laser trabeculoplasty versus argon laser trabeculoplasty in pseudophakic glaucoma patients. | Clin Ophthalmol 2012; 6: 1935-40. | SLT versus SLT |
| 3 | Liu Y, Birt CM. | Argon versus selective laser trabeculoplasty in younger patients: 2-year results. | J Glaucoma 2012; 21(2): 112-5. | SLT versus SLT |
| 4 | Bovell AM, et al. | Long term effects on the lowering of intraocular pressure: selective laser or argon laser trabeculoplasty? | Can J Ophthalmol 2011; 46(5): 408-13. | SLT versus SLT |
| 5 | Almeida EJ, et al. | Pattern of intraocular pressure reduction following laser trabeculoplasty in open-angle glaucoma patients: comparison between selective and nonselective treatment. | Clin Ophthalmol 2011; 5: 933-6. | SLT versus other laser |
| 6 | Russo V, et al. | Selective laser trabeculoplasty versus argon laser trabeculoplasty in patients with uncontrolled open-angle glaucoma. | Eur J Ophthalmol 2009; 19(3): 429-34. | SLT versus SLT |
| 7 | Birt CM, et al. | Selective laser trabeculoplasty retreatment after prior argon laser trabeculoplasty: 1-year results. | Can J Ophthalmol 2007; 42(5): 715-9. | Not naïve patients |
| 8 | Best UP, et al. | Pressure reduction after selective laser trabeculoplasty with two different laser systems and after argon laser trabeculoplasty--a controlled prospective clinical trial on 284 eyes. | Klin Monbl Augenheilkd 2007; 224(3): 173-9. | Not naïve patients |
| 9 | Damji KF, et al. | Selective laser trabeculoplasty versus argon laser trabeculoplasty: results from a 1-year randomised clinical trial. | Br J Ophthalmol 2006; 90(12): 1490-4. | SLT versus SLT |
| 10 | Van de Veire S, et al. | Argon versus selective laser trabeculoplasty. | Bull Soc Belge Ophtalmol 2006;(299): 5-10. | SLT versus SLT |
| 11 | Zeyen T, et al. | Baseline IOP predicts selective laser trabeculoplasty success at 1 year post-treatment: results from a randomised clinical trial. | Br J Ophthalmol 2005; 89(9): 1157-60. | SLT versus SLT |
| 12 | Juzych MS, et al. | Comparison of long-term outcomes of selective laser trabeculoplasty versus argon laser trabeculoplasty in open-angle glaucoma. | Ophthalmology 2004; 111(10): 1853-9. | SLT versus SLT |
| 13 | Martinez-de-la-Casa JM, et al. | Selective vs argon laser trabeculoplasty: hypotensive efficacy, anterior chamber inflammation, and postoperative pain. | Eye (Lond) 2004; 18(5): 498-502. | SLT versus SLT |
| 14 | Popiela G, et al. | Use of YAG-Selecta laser and argon laser in the treatment of open angle glaucoma. | Klin Oczna 2000; 102(2): 129-33. | SLT versus SLT |
| 15 | Damji KF, et al. | Selective laser trabeculoplasty v argon laser trabeculoplasty: a prospective randomised clinical trial. | Br J Ophthalmol 1999; 83(6): 718-22. | SLT versus SLT |
| 16 | Hollo G. | Argon and low energy, pulsed Nd:YAG laser trabeculoplasty. A prospective, comparative clinical and morphological study. | Acta Ophthalmol Scand 1996; 74(2): 126-31. | SLT versus SLT |
| 17 | Wang QY, et al. | Research review of selective laser trabeculoplasty for open angle glaucoma. | International Journal of Ophthalmology 2011; 11(5): 828-9. | Review |
| 18 | Liu H, et al. | Clinical observation of selective laser trabeculoplasty on primary open angle glaucoma. | Chinese Journal of Practical Ophthalmology 2011; 29: 385-7. | SLT versus SLT |
| 19 | Zhou M, et al. | Selective Laser Trabeculoplasty on Early Primary Open Angle Glaucoma: a Clinical Research. | Chinese Journal of Laser Medicine and Surgery 2011; 20: 230-3. | SLT versus SLT |
| 20 | Zhou W, et al. | A retrospective study of selective laser trabeculoplasty in the treatment of primary open angle glaucoma. | Chinese Journal of Practical Ophthalmology 2011; 29: 830-2. | Not prospective study |
| 21 | LI D, et al. | Intraocular pressure alterations around selective laser trabeculoplasty in primary open-angle glaucoma patients. | Chinese Journal of Practical Ophthalmology 2009; 27: 1291-2. | SLT versus SLT |
| 22 | Qian SH, Sun XH. | Selective laser trabeculoplasty in the treatment of primary open-angle glaucoma. | Zhonghua Yi Xue Za Zhi 2007; 87(2): 118-20. | SLT versus SLT |
| 23 | Ma Y, et al. | Effect of Selective Laser Trabeculoplasty on Primary Open Angle Glaucoma. | Chinese Journal of Laser Medicine and Surgery 2006; 15: 307-9. | SLT versus SLT |
| 24 | Yuan R, et al. | The therapeutic effect of selective laser trabeculoplasty to intraocular hypertension of open angle glaucoma after trabeculectomy. | Chinese Journal of Practical Ophtahalmology 2006; 2: 46-8. | Not naïve patients |
| 25 | Chen L, et al. | Acute histopathological changes after two types of trabeculoplasty with different wavelength laser: A Comparative Study. | Chinese Journal of Laser Medicine and Surgery 2002; 11: 161-5. | SLT versus SLT |
